# Imaging White Matter in Human Brainstem

**DOI:** 10.3389/fnhum.2013.00400

**Published:** 2013-07-24

**Authors:** Anastasia A. Ford, Luis Colon-Perez, William T. Triplett, Joseph M. Gullett, Thomas H. Mareci, David B. FitzGerald

**Affiliations:** ^1^Department of Veterans Affairs Rehabilitation Research and Development Brain Rehabilitation Research Center, Malcom Randall VA Medical Center, Gainesville, FL, USA; ^2^Department of Psychology, University of Florida, Gainesville, FL, USA; ^3^Department of Physics, University of Florida, Gainesville, FL, USA; ^4^Department of Biochemistry and Molecular Biology, University of Florida, Gainesville, FL, USA; ^5^Department of Clinical and Health Psychology, University of Florida, Gainesville, FL, USA; ^6^Neurology Service, North Florida/South Georgia Veterans Health System, Gainesville, FL, USA; ^7^Department of Neurology, University of Florida, Gainesville, FL, USA

**Keywords:** diffusion-weighted imaging, tractography, brainstem, white matter, high-resolution MRI

## Abstract

The human brainstem is critical for the control of many life-sustaining functions, such as consciousness, respiration, sleep, and transfer of sensory and motor information between the brain and the spinal cord. Most of our knowledge about structure and organization of white and gray matter within the brainstem is derived from *ex vivo* dissection and histology studies. However, these methods cannot be applied to study structural architecture in live human participants. Tractography from diffusion-weighted magnetic resonance imaging (MRI) may provide valuable insights about white matter organization within the brainstem *in vivo*. However, this method presents technical challenges *in vivo* due to susceptibility artifacts, functionally dense anatomy, as well as pulsatile and respiratory motion. To investigate the limits of MR tractography, we present results from high angular resolution diffusion imaging of an intact excised human brainstem performed at 11.1 T using isotropic resolution of 0.333, 1, and 2 mm, with the latter reflecting resolution currently used clinically. At the highest resolution, the dense fiber architecture of the brainstem is evident, but the definition of structures degrades as resolution decreases. In particular, the inferred corticopontine/corticospinal tracts (CPT/CST), superior (SCP) and middle cerebellar peduncle (MCP), and medial lemniscus (ML) pathways are clearly discernable and follow known anatomical trajectories at the highest spatial resolution. At lower resolutions, the CST/CPT, SCP, and MCP pathways are artificially enlarged due to inclusion of collinear and crossing fibers not inherent to these three pathways. The inferred ML pathways appear smaller at lower resolutions, indicating insufficient spatial information to successfully resolve smaller fiber pathways. Our results suggest that white matter tractography maps derived from the excised brainstem can be used to guide the study of the brainstem architecture using diffusion MRI *in vivo*.

## Introduction

The brainstem is a complex neural structure crucial for sustaining survival functions. These functions include respiration, cardiovascular regulation, sleep, consciousness, and transmission of sensory and motor information between the brain and the spinal cord (Nicholls and Paton, 2009). Knowledge of the structural organization in the brainstem originates from animal and human dissection and histology studies, and, in the more recent years, from human neuroimaging studies (Harting, 1977; Steriade et al., 1988; Stieltjes et al., 2001; Habas and Cabanis, 2007; Naidich et al., 2008; Kamali et al., 2010).

High-resolution magnetic resonance imaging (MRI) can be used to visualize the brainstem *in vivo*. Unfortunately, the brainstem is quite dense functionally with many nuclei and small cortical and spinal white matter projections. These structures can be difficult to visualize on standard clinical MRI scans as there is not sufficient tissue-specific contrast. Recently developed diffusion-weighted imaging (DWI) is a non-invasive technique that allows visualization of gray and white matter architecture *in vivo*. In particular, DWI tractography can be used to infer white matter pathways based on the properties of water diffusion within underlying tissue. This method provides many valuable insights into white matter organization within the brain and also has been applied to visualize brainstem neural architecture (Nagae-Poetscher et al., 2004; Behrens et al., 2007; Habas and Cabanis, 2007; Catani and Thiebaut de Schotten, 2008; Rilling et al., 2008).

The anatomical complexity and location of the brainstem, however, presents a number of methodological challenges for *in vivo* tractography. In particular, pulsatile and respiratory motion resulting from the cardiac cycle and respiratory activity may produce significant distortions of the brainstem tissue during image acquisition. Cerebrospinal fluid (CSF) has been found to flow in both rostral and caudal directions during the cardiac cycle, with velocity through the aqueduct of Sylvius of roughly 14 mm/s in the cranial to caudal direction and −12 mm/s caudal to cranial (Sweetman and Linninger, 2011). This cardiac synchronized flow in the CSF can result in brainstem pulsations. Two different techniques have been suggested for reducing the distortions due to these pulsations. One, using a navigator-corrected approach results in reduced distortions, without complete elimination of cardiac pulsation (Jiang et al., 2002). The second is to use cardiac gating, which reduces distortion, although with longer acquisition times (Jiang et al., 2002). These techniques may not fully correct for pulsatile motion and may result in incomplete white matter modeling.

The present study investigates white matter organization within the brainstem using an excised tissue sample. This approach avoids motion-related artifacts and allows the limits of resolution to be studied by acquiring diffusion-weighted data at higher resolutions and magnetic field strengths than those currently possible *in vivo*. In addition, to examine how resolution affects the visualization of brainstem white matter pathways is altered as a function of data resolution, we acquired two additional diffusion-weighted MRI datasets of the same tissue using lower acquisition resolutions. These lower resolutions corresponded to the highest resolution currently available for whole brain *in vivo* acquisitions (1 mm isotropic), and to the most commonly used acquisition resolution in whole brain *in vivo* acquisitions (2 mm isotropic).

## Materials and Methods

### The brainstem tissue

**Figure 1:**
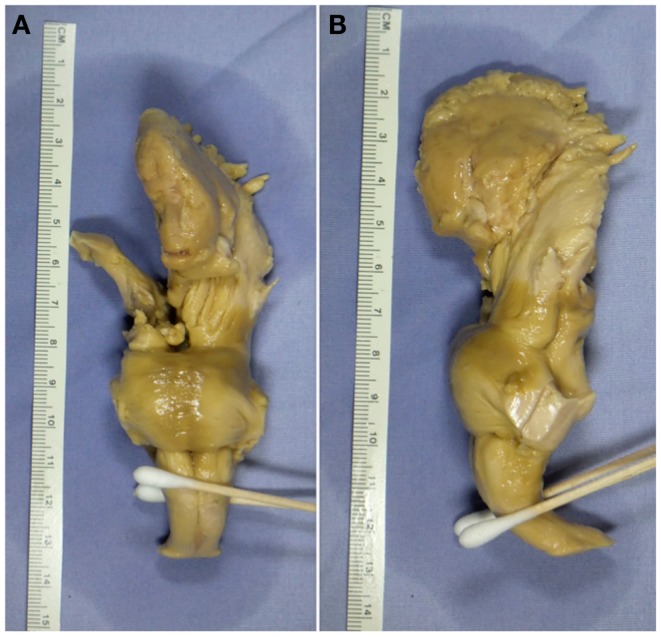
***Ex vivo* tissue sample**. **(A)** Coronal view displaying the anterior portion of the tissue sample. **(B)** Sagittal view displaying the left-hand side of the tissue sample.

The brain, including brainstem was obtained from the University of Florida Neuromedicine Human Brain Tissue Bank. The donor was a 78-year-old male who died of a cerebrovascular accident. The post-mortem interval prior to fixation was 18 h. The tissue had been fixed in 10% formalin after removal (Figure 1) and, prior to MRI measurement, was then washed in phosphate-buffered saline (PBS) to remove formalin. Written informed consent was obtained post-mortem from the patient’s son in compliance with Institutional Review Board guidelines of the University of Florida and North Florida/South Georgia Malcom Randall Veteran’s Affairs Medical Center.

### Image acquisition

Prior to MR data acquisition, the brainstem was washed for 5 days in PBS, with periodic solution exchanges, then placed in a 41 mm plastic cylinder filled with *Fluorinert* (3M, St. Paul, MN, USA). The brainstem was imaged with a pulsed field-gradient, spin echo sequence at 470 MHz in a 11.1 T 40 cm horizontal bore imaging spectrometer (Agilent Technologies, Santa Clara, CA, USA) using a custom-built volume transmit/receive coil. Three image datasets were acquired with isotropic resolution of (1) 0.333 mm, using a frequency by phase encode matrix of in 114 slices, (2) 1.0 mm, using a frequency by phase encode matrix in 40 slices, and (3) 2.0 mm, using a frequency by phase encode matrix of in 20 slices. The images were obtained with repetition times (TR’s) of 5110.9, 4000, and 3000 ms for 0.333, 1, and 2 mm resolution, respectively, with an echo time (TE) of 31 ms, using two signal averages, gradient pulses of 4 ms width, and intervals of 18 ms between gradient pulses. The TE value was kept constant across the three acquisition resolutions to ensure that T2 effects did not differ across the acquisitions and did not result in signal-to-noise ratio (SNR) differences. The total field-of-view for each acquisition was 70 mm × 40 mm × 38 mm. Low diffusion-weighted images (100 s/mm^2^) were acquired in six directions, and high-diffusion-weighted images (1250 s/mm^2^) were acquired in 64 directions distributed uniformly over a sphere following an electrostatic repulsion scheme (Jones et al., 1999).

The above image acquisition approach results in different SNRs for the three datasets. The lowest SNR was obtained with the 0.333 mm isotropic resolution data, where the SNR for low diffusion weighting (100 s/mm^2^) is ∼32, and 25 for high-diffusion-weighted data (1250 s/mm^2^). As suggested in a recommendation that SNR should be above 20 to assure stable tensor estimations (Bastin et al., 1998), each dataset should contain sufficient SNR so that the contrast-to-noise ratio is suitable for stable tensor estimates and to produce consistent streamline pathways. In addition, the sampling scheme (Jones et al., 1999) used in this study and number of gradient directions also improve the tensor estimates (Batchelor et al., 2003).

Although the TE was held fixed, the TRs were shortened with decreasing resolution in order to reduce the total acquisition time for the three data sets (∼38 h). Therefore the longitudinal relaxation time (T1) weighting differed slightly between the three data sets. However, the minimum TR used in this study was still longer than brain T1 values previously reported (de Graaf et al., 2006; Oros-Peusquens et al., 2008; Wright et al., 2008). In particular, Wright and colleagues reported T1 relaxation times in human brain of 1.12 and 1.93 s for white and gray matter respectively at 7 T, and de Graaf and colleagues reported rat brain white and gray matter values of 1.75 and 2.11 s respectively at 11.7 T. Since T1 relaxation times in tissue appear to increase logarithmically with magnetic field strength, the measured human brain T1’s at 7 T can be projected to be appropriately between 1.3 and 2.2 s at 11.1 T. Also the excised fixed brain stem used in this study was immersion fixed with formalin after an unknown post-mortem interval (typically hours). Formaldehyde fixation is known to shorten T1 by as much as 21% (Shepherd et al., 2009b) and T1 is known to increase with post-mortem interval with a 20% increase after 24 h (Shepherd et al., 2009a). Therefore, the effect of fixation and post-mortem on T1 may be assumed to balance. Using the estimated 11.1 T values of *in vivo* human brain T1’s, the signal strength would be reduced by ∼12% between the longest TR (∼5 s for 0.333 mm resolution) and shortest TR (3 s for 2 mm resolution) TR. Thus, the effect of T1 relaxation would not appear to have significant effect on the estimated streamline tractography.

### Image processing

MR data was processed with in-house software written in Interactive Data Language (IDL; Exelis Visual Information Systems, Boulder, CO, USA). The image intensity attenuation for each voxel was fitted as a linear decay to a rank-2 tensor dependent on the diffusion weightings, then the average diffusivity (AD) and fractional anisotropy (FA) values were calculated from the resulting tensor at each voxel (Basser and Jones, 2002). To infer fiber tract streamlines, the displacement probability of water self-diffusion in each voxel is estimated using the Mixture of Wishart method distributions (Jian and Vemuri, 2007) for an average diffusion displacement of 6 μm then the maximum displacement probability within tissue in each voxel is identified. This method allows the tracking of crossing and branching fibers, and makes the current algorithm superior to traditional streamline tracking techniques based on the rank-2 tensor models (Basser et al., 1994; Jian et al., 2007). Tractography is performed by seeding each voxel in the brain with a sub-voxel grid with evenly spaced seed points. From each seed point, one streamline is launched bi-directionally for each estimated displacement probability maximum contained in that voxel using the FACT algorithm (Mori et al., 1999). Each streamline front is propagated by stepping 0.5 voxel width in the direction of the maximum that is most inline with the streamline’s present direction of travel. In order to prevent streamlines from looping back, angular deviation of the track is limited to 65°. If the estimated track exceeds this threshold, the streamline is stopped.

In order to determine a seeding density that would produce consistent results across the three acquisition resolutions, each dataset was seeded with 8, 27, and 64 seed points per voxel, then the percent change in edge weight (see below) was compared across the three seeding densities for each acquisition (Table 1). Edge weight is a dimensional quantity that represents tract connectivity strength, independent of acquisition resolution and seeding density. Our results for the 0.333 mm isotropic dataset showed that the percent change in edge weight values for all pathways differed by less than 7% as we increased the seeding density from 8 to 27 seeds per voxel. As seeding density was increased from 27 to 64 seeds per voxel, the percent change was less than 2% for all pathways (Table 1). For the 1 mm isotropic dataset, the percent change in edge weights was less than 3% for the corticopontine/corticospinal, and for the middle cerebellar peduncle (MCP) pathways as seeding density was increased from 8 to 27 and also from 27 to 64 seeds per voxel. The percent change for the superior cerebellar peduncle pathways was 32.49% as seeding density was increased from 8 to 27 seeds, and 19.88% as seeding density was increased from 27 to 64 seeds per voxel (Table 1). Due to this large difference in edge weight values, we seeded the 1 mm isotropic dataset using 125 seeds per voxel and computed the edge weight value for the superior cerebellar peduncle pathways. Edge weight value for this seeding density differed by less than 1% from the edge weight value computed by the 64 seeds per voxel seeding density. Seeding density results for the 2 mm isotropic dataset showed that for all pathways the edge weight values differed by less than 6% as we changed the seeding density from 8 to 27 seeds and from 27 to 64 seeds per voxel (Table 1). Overall, these results show that seeding density of 64 seeds per voxel provide a stable result, so the tractography results presented below are pathways traced using a track seeding density of 64 seeds per voxel.

**Table 1:** **Percent change in edge weights as a function of seed density**.

Acquisition resolution (mm isotropic)	% Change in edge weight as seed density changes from 8 to 27 seeds per voxel	% Change in edge weight as seed density changes from 27 to 64 seeds per voxel	% Change in edge weight as seed density changes from 64 to 125 seeds per voxel
0.333	7	2	–
1	3 (32.49 for SCP pathways)	3 (19.88 for SCP pathways)	– (1 for SCP pathways)
2	6	6	–

*Values listed in the table represent percent change in edge weight values for corticopontine/corticospinal and middle cerebellar peduncle pathways. Superior cerebellar peduncle pathways edge weight percent changes are listed in parenthesis*.

These image processing methods were applied to each of the acquired scans (333.3 μm, 1, and 2 mm isotropic). To determine how resolution effects the inference of fiber pathways, we compared trajectories and diffusion characteristics for pathways of interest across the three acquisition resolutions from the best available *ex vivo* resolution (0.333 mm isotropic) to the best possible resolution *in vivo* (1 mm isotropic), and to a commonly used *in vivo* resolution (2 mm isotropic) (see Figures 3–5 below).

### Regions of interest

In order to define representative white matter pathways within the excised tissue, we used Duvernoy’s atlas (Naidich et al., 2008) to create six regions of interest (ROI’s) to define the boundaries of four white matter fiber pathways; (1) corticopontine/corticospinal (CST/CPT) fibers, (2) fibers passing through the superior cerebellar peduncle (SCP), (3) fibers passing through the MCP, and (4) the medial lemniscus (ML) pathways. The regions were drawn on the FA map, which provides good white/gray matter contrast.

**Figure 2:**
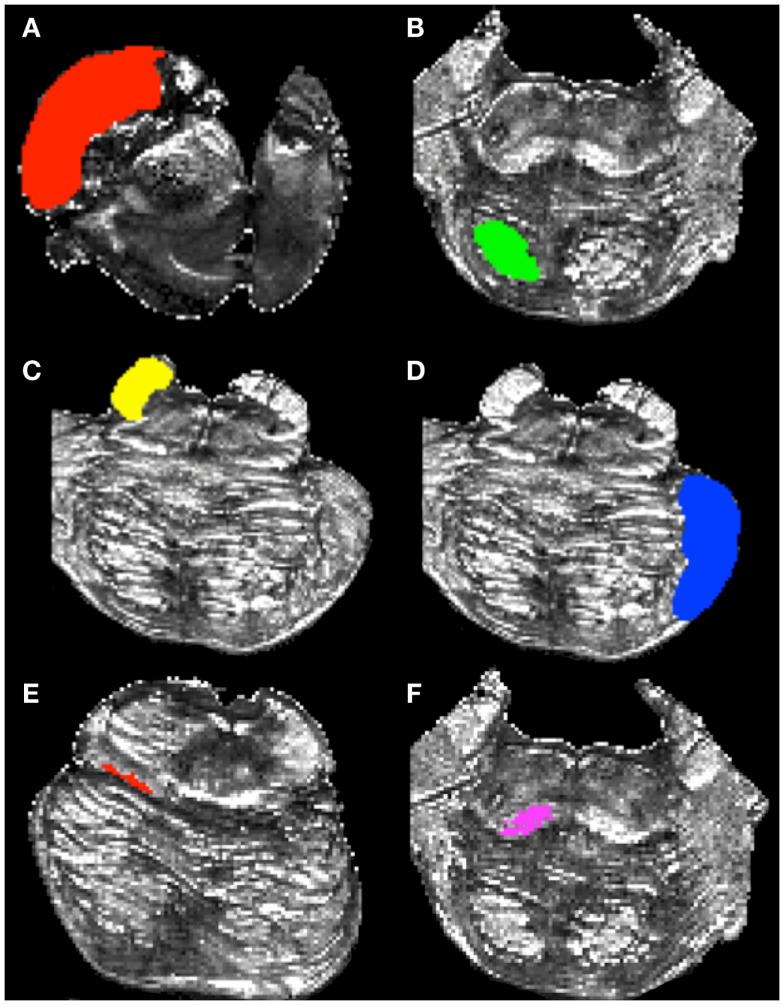
**Regions of interest used to delineate white matter pathways within the brainstem**. **(A)** cerebral peduncle mask, **(B)** inferior pons mask, **(C)** superior cerebellar peduncle (SCP) mask, **(D)** middle cerebellar peduncle mask (MCP), **(E)** superior medial lemniscus (ML) mask, and **(F)** inferior ML mask.

To delineate the CS/CP pathway, we created two ROI’s within the pons. The first delineated the cerebral peduncle and is drawn within the superior-most axial slice where this structure can be clearly identified. We delineated the cerebral peduncle within this slice and six additional slices inferior to it, creating a seven-slice ROI (Figure 2A). The inferior border of the second ROI was located within the inferior-most axial slice of the pons and followed six slices superior to it, creating a seven-slice ROI (Figure 2B).

To create the ROI’s to identify the SCP fibers (Figure 2C), we first located the superior-most axial slice where the SCP could be identified definitively. Next, we identified the most inferior portion of the peduncle and recorded the corresponding axial slice number. We then computed the axial slice number corresponding to a mid-way point between the superior and inferior borders of the SCP. Lastly, we outlined the peduncle within the mid-point axial slice and the three slices inferior and superior to it, creating a seven-slice ROI. In addition, we created a second, or waypoint, ROI to infer cerebellar efferent traveling through the SCP toward the thalamus and the cortex. The waypoint ROI was located contralateral to the SCP ROI, and was drawn on the same axial slices as the cerebral peduncle ROI to outline the remainder of the brainstem tissue outside of the cerebral peduncle.

The MCP ROI was drawn following the steps described below. First, we identified the most superior axial slice corresponding to the superior border of the MCP. Next, we identified the inferior extent of the MCP and recorded the corresponding axial slice number. We then computed the axial slice number corresponding to the mid-point between the superior and inferior extents of the MPC. We outlined the MCP within that slice and the three slices superior and inferior to it creating a seven-slice ROI (Figure 2D). We used the cerebral peduncle ROI (Figure 2A) as a waypoint to infer pathways traveling to the cerebellum from the corticopontine nuclei and the cortex. The cerebral peduncle ROI was drawn on the contralateral side of the brainstem tissue.

To delineate the ML pathways we created two ROIs. The first ROI was drawn on an axial slice corresponding to the superior extent of the pathway determined by examining the sagittal view of the FA image, as well as on nine consecutive inferior axial slices (Figure 2E). The final ROI included voxels comprising the ML pathway within each of the 10 axial slices. The second ROI was drawn on an axial slice corresponding to the inferior extent of the ML pathway identified by the sagittal view of the FA map, as well as on nine superior axial slices (Figure 2F). The second ROI consisted of 10 axial slices and included voxels comprising ML pathways within each of these slices. To ease the tracking process we chose to include more axial slices in the ML ROIs as compared to the remaining ROIs because this pathway is smaller than the other three pathways we examined.

All ROI’s were drawn on an FA image derived from the highest resolution diffusion scan (0.333 mm) and registered to 1, 2 mm scans using linear registration with nearest-neighbor interpolation using FSL FLIRT (Jenkinson and Smith, 2001). Table 2 lists surface area values for each of these ROI in all five datasets. Surface area measurements are used in edge weight value calculations (see Eq. 1 below).

**Table 2:** **Surface areas of regions of interest used to infer white matter pathways**.

Resolution (mm^3^)	Superior pons (mm^2^)	Inferior pons (mm^2^)	Middle cerebellar peduncle (mm^2^)	Superior cerebellar peduncle (mm^2^)	Waypoint mask for superior cerebellar peduncle pathways (mm^2^)	Superior medial lemniscus (mm^2^)	Inferior medial lemniscus (mm^2^)
0.333	404.34	110.64	314	118.22	581.68	78.22	113.47
1	343.7	98.87	314.66	117.36	300.24	74.49	71.96
2	388.45	112.87	358.49	116.68	289.82	27.71	55.43

### Tractography analysis

To infer the corticopontine/corticospinal tracts (CPT/CST) pathways, we filtered whole brainstem streamline tractography results to include only fibers that intersect the cerebral peduncle ROI then filtered these fiber pathways further with the inferior pons ROI so only pathways connecting both ROI’s were kept for further analysis. In order to delineate the SCP pathways, we filtered whole brainstem streamline tractography results with the SCP ROI to infer pathways passing through this region. Next, we filtered the resulting pathways with the waypoint ROI to infer cerebellar efferent fibers traveling toward the thalamus and the cortex. Similarly, to infer MCP pathways we first filtered whole brainstem streamline tractography results with the MCP ROI. Then we applied the cerebral peduncle ROI as a waypoint to visualize cerebellar afferents traveling from the cortex and pontine nuclei toward the cerebellum. The ML pathways were inferred by first intersecting the whole brainstem tractography results with the superior ML ROI, and then further filtering these results by applying the inferior ML ROI.

In order to visualize how the appearance of the streamline pathways is changed as a function of acquisition resolution, we created overlap images of the four pathways inferred at three acquisition resolutions (Figure 7). Specifically, after delineating each of the four streamline pathways in each dataset, we registered the resulting streamline pathways from the 1 to 2 mm datasets to the 0.333 mm dataset using linear nearest-neighbor transformation using FSL FLIRT (Jenkinson and Smith, 2001). The streamline pathways were binarized; if a voxel was occupied by a streamline pathway, it was assigned a value of 1. If a voxel was not occupied by a streamline pathway, it was assigned a value of 0. The binarized streamline pathways (0.333 mm pathways, 1 mm pathways registered to 0.333 mm, and 2 mm pathways registered to 0.333 mm) were combined into a single pathway by summing the voxel values. If a voxel belonged to a given streamline pathway in all three datasets this voxel would be assigned a value of 3, if only two datasets had this voxel in common it would be assigned a value of 2. Similarly, if a voxel was unique to one of the datasets, it would be assigned a value of 1. This approach allowed us to examine the spatial variability of the inferred streamline pathways as a function of acquisition resolution. In addition, we calculated percent overlap between the 0.333 mm streamlines and registered 1 and 2 mm streamlines (Table 4).

### Quantitative tractography measures

After calculating the streamline tracts along these pathways, we quantified the strength of fiber connectivity for the resulting tracts using measures of tract volume and edge weight. Tract volume represents the number of voxels occupied by each of the streamline tracts multiplied by the volume of each voxel (measured in mm^3^) and the edge weight represents strength of connectivity of each pathway and is derived as an application of graph theory (Hagmann et al., 2008; Colon-Perez et al., 2012).

Edge weight, *w*(*e_ij_*), is a scalar, dimensionless quantity independent of the acquisition resolution and seeding density. This measure is computed for inferred fiber tracts between two ROI (Eq. 1 below). From graph theory perspective, each ROI defines a network node (e.g., *n_i_* or *n_j_*) and each pathway connecting a pair of nodes, *n_i_* and *n_j_*, represents an network edge (e.g., *e_ij_*). In Eq. 1, the edge weight is dependent on the inverse sum of the streamline path length, *l*(*f_p,m_*), between the nodes. The inner summation involves all seed points (*P*_voxel_) in each voxel (*p* = *1* to *P*_voxel_) that belong the edge. The outer summation involves all voxels (*M*) in the edge (*m* = *1* to *M*), connecting the nodes *n_i_* and *n_j_*, that originate only from voxels in the streamline path connecting the nodes (located at *x_p,m_*, *y_p,m_*, and *z_p,m_*).

(1) $$\begin{array}{llll} w\left(e_{\mathrm{ij}}\right) & =\left(\frac{V_{\text{voxel}}}{P_{\text{voxel}}}\right)\left(\frac{2}{A_{i}+A_{j}}\right)\; & & \;\\ & \;\times \overset{M}{\underset{m=1}{\sum }}\overset{P}{\underset{p=1}{\sum }}\frac{\delta \left(x-x_{p,m}\right)\delta \left(y-y_{p,m}\right)\delta \left(z-z_{p,m}\right)}{l\left(f_{p,m}\right)}\; \end{array}$$

This double sum over the inverse path length [1/*l*(*f_p,m_*)] effectively provides a count of the number of unique streamlines in the pathway, which is not dependent on the length of the streamlines.

Terms to the left of the double sum represent scaling factors that result in a dimensionless quantity. Specifically, *A_i_* and *A_j_* are surface areas of the two ROI defining the nodes. Scaling by the average of these surface areas allows direct comparison of edge weights for pathways with various size ROI. Multiplying by the ratio of voxel volume, V_voxel_, and the number of seed points, P_voxel_, represents a normalization by the seed point density used during the streamline tractography analysis to ensure that the edge weight is independent of the acquisition resolution and seed density. This approach allows us to compare edge weights for data acquired using different acquisition resolutions and analyzed using different number of seeds per voxel.

## Results

Using our tractography approach we delineated the corticopontine/corticospinal pathways, MCP pathways, superior cerebellar peduncle pathways, and ML pathways. Table 3 lists track volumes and edge weight values for each of these pathways inferred from the three acquisition resolution datasets (0.333, 1, and 2 mm isotropic). Overall, we note that tract volumes and edge weight values of the fiber bundles increase in magnitude as acquisition resolution is decreased from 0.333 to 2 mm (with exception of the superior cerebellar pathways where tract volume and edge weight are smaller for 1 mm dataset as compared with 0.333 and 2 mm results and the ML pathways where tract volume and edge weight for the 2 mm dataset are smaller than for the 0.333 and 1 mm datasets.). Table 2 indicates that surface areas of ROI used to infer the pathways did not vary significantly as a function of data resolution (with the exception of the ML ROI). This observation implies that larger track volumes and edge weight values observed in lower acquisition resolution datasets did not result from volume changes in the ROI. Rather, as resolution is decreased from 0.333, to 1 and 2 mm resolution, volume averaging effects artificially inflate track volumes and edge weight values as extraneous pathways are included along with the pathways of interest. Larger acquisition voxels provide less information about branching and crossing fibers as well as about tissue-type boundaries. This is particularly important for brainstem anatomy as gray matter nuclei and their white matter projections are dense and relatively small in size.

**Table 3:** **Tract volumes and edge weight values of the brainstem white matter pathways inferred from three acquisition resolution datasets**.

Resolution (mm^3^)	CPT/CST^a^		SCP^b^		MCP^c^		ML^d^	
	Tract volume (mm^3^)	Edge weight	Tract volume (mm^3^)	Edge weight	Tract volume (mm^3^)	Edge weight	Tract volume (mm^3^)	Edge weight
0.333	1789.4	3.15e^−2^	749.6	5.57e^−4^	1949.5	1.45e^−3^	530	9.76e^−2^
1	2556	7.53e^−2^	485	2.15e^−4^	3776	3.81e^−2^	531	8.17e^−2^
2	2472	1.18e^−1^	704	8.25e^−4^	3728	3.72e^−2^	272	4.26e^−3^

*^a^Corticopontine/corticospinal tract; ^b^superior cerebellar peduncle tracts; ^c^middle cerebellar peduncle tracts; ^d^medial lemniscus tracts*.

We note that the surfaces areas of the ROI used to delineate the ML pathways are smaller in 1 and 2 mm datasets as compared to the 0.333 mm dataset (Table 2). This finding seems to suggest that due to the effects of volume averaging, these lower resolution scans do not contain enough spatial information to successfully identify voxels within small white matter pathways such as the ML. This effect greatly limits the size of the pathways that can be successfully resolved at these lower resolutions, as the ROIs necessary to delineate the pathways are difficult to identify accurately, especially in the 2 mm dataset.

### Corticopontine/corticospinal pathways

**Figure 3:**
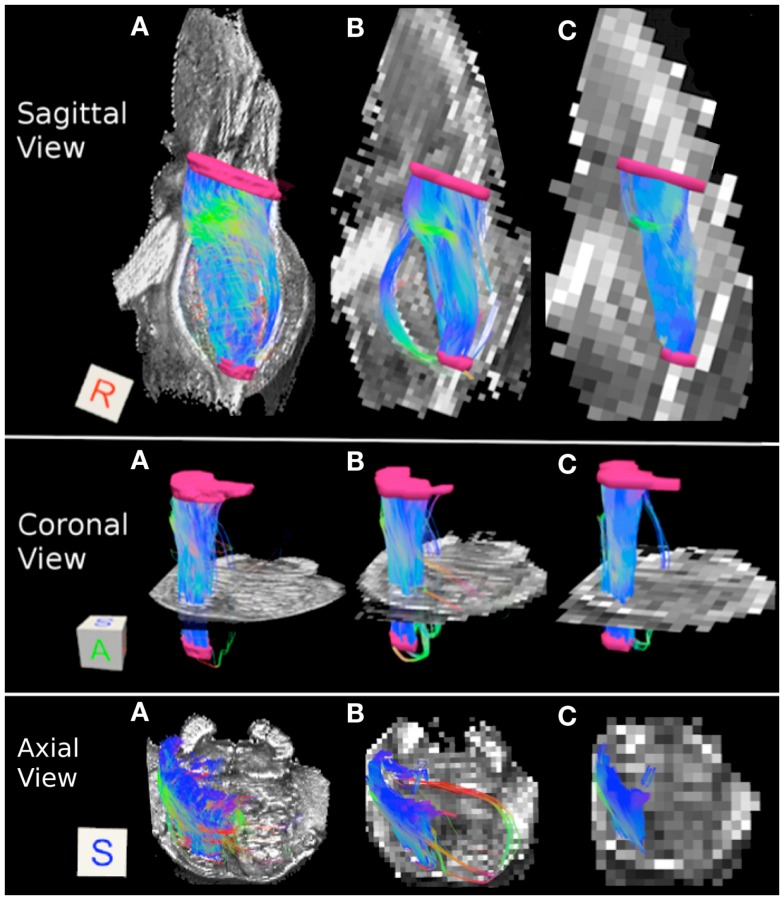
**Corticopontine/corticospinal pathways: (A) 0.333 mm isotropic acquisition resolution, (B) 1 mm resolution, (C) 2 mm resolution**. Top row: sagittal view of the pathways with a mid-sagittal slice of the FA maps serving as a background. Middle row: coronal view of the pathways with an axial slice of the FA maps serving as a background. Bottom row: axial view of the pathways with an axial slice of the FA maps serving as a background. Regions of interest used to delineate the pathways (cerebral peduncle and inferior pons) depicted in pink. The regions of interest are not included in the axial view images for ease of visualization of the pathways. Three-dimensional cubes to the right of the images represent spatial rotations of the pathways, where faces of the cubes represent orientations of the *x*, *y*, and *z* axes in space: R (right, *x*-axis), A (anterior, *y*-axis), S (superior, *z*-axis). Color gradient within the pathways represent local fiber orientation: red – medial/lateral; blue – superior/inferior; green – anterior/posterior.

Figure 3 depicts the corticopontine/corticospinal pathways inferred from the three datasets with different acquisition resolutions and from the interpolated datasets. Corticopontine/corticospinal pathways inferred from the 0.333 mm isotropic dataset connect the cerebral peduncle with the inferior pons. The streamline pathway occupies most of the anterior-posterior extent of the pons. In addition, a small secondary streamline pathway can be seen on the sagittal view of the pathways (Figure 3A, top row).

Corticopontine/corticospinal pathways inferred in the 1 mm dataset also connect the cerebral peduncle and the inferior pons ROI’s. In addition, and unlike the 0.333 mm dataset, an additional, secondary streamline courses within the posterior portion of the pons (Figure 3B, top row). This secondary streamline likely includes a small portion of the ML and/or pathways within the anterior lateral system (ALS), due to its close spatial proximity to the inferior pons ROI (Haines, 2004). The inferred streamline pathways also appear to include a small portion of the fibers that course medially and cross over to the contralateral side of the brainstem (Figure 3B, bottom row). These contralateral pathways are likely to be the cerebellar afferents that pass through the cerebral peduncle and cross over to the contralateral MCP. Increase prevalence of extraneous streamline pathways, observed in the 1 mm dataset, is likely the result of increased volume averaging present in this lower resolution scan.

Figure 3C depicts corticopontine/corticospinal streamline pathways inferred in the 2 mm dataset. Overall the majority of the pathways look consistent with those inferred from the 0.333 and 1 mm datasets. Interestingly, the extraneous pathways, likely to be comprised by the ML/ALS fibers observed in the 1 mm dataset, are not present in the 2 mm dataset. The 2 mm resolution dataset does contain extraneous streamline pathways, but they are located within the posterior medial portion of the pons and are likely to be the medial longitudinal fasciculus (MLF) tracts (Figure 3C bottom row).

### Superior cerebellar peduncle

**Figure 4:**
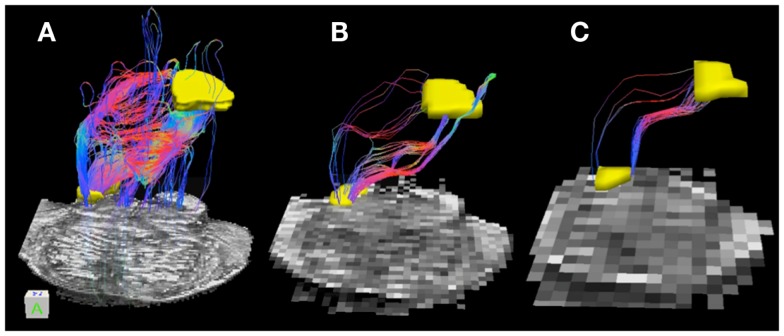
**Superior cerebellar peduncle pathways: (A) 0.333 mm isotropic acquisition resolution dataset, (B) 1 mm isotropic resolution dataset, (C) 2 mm isotropic resolution dataset**. An axial slice of the FA maps serves as a background image. Superior cerebellar peduncle (SCP) and a waypoint mask are depicted in yellow. Color gradient and three-dimensional cube colors are the same as in Figure 3.

Table 3 lists tract volumes and edge weight values for the superior cerebellar peduncle streamline pathways inferred from the three datasets with different acquisition resolutions and from the interpolated datasets. Both the track volume and edge weight value for the 0.333 and the 2 mm datasets are larger in magnitude than those for the 1 mm dataset. Figure 4 depicts the superior cerebellar streamline pathways inferred in 0.333, 1, and 2 mm datasets.

Superior cerebellar peduncle streamline pathways, that carry cerebellar efferents toward the thalamus and cortex and traveling through the SCP, cross over to the contralateral side of the brainstem within the cerebellar peduncle decussation (indicated by red color within the streamline pathways in Figure 4), and ascend further within the superior extent of our brainstem sample. We observe that these streamline pathways appear to include some extraneous tracts in the 0.333 mm dataset, where these additional pathways descend down into the contralateral side of the brainstem (Figure 4A). These extraneous pathways are absent in the 1 mm dataset, where a vast majority of the pathways follow known anatomical trajectory of the cerebellar efferents (Haines, 2004). The majority of the SCP streamline pathways within the 2 mm dataset originate in the medial extent of the SCP, allowing us to visualize only a fraction of the SCP pathways (Figure 4C).

### Middle cerebellar peduncle

Table 3 indicates that the MCP streamline pathways, that carry cerebellar afferents from the thalamus and cortex, increase in volume and have larger edge weight values as resolution decreases. Specifically, the 0.333 mm isotropic dataset has the smallest tract volume, followed by the 2 and 1 mm datasets.

**Figure 5:**
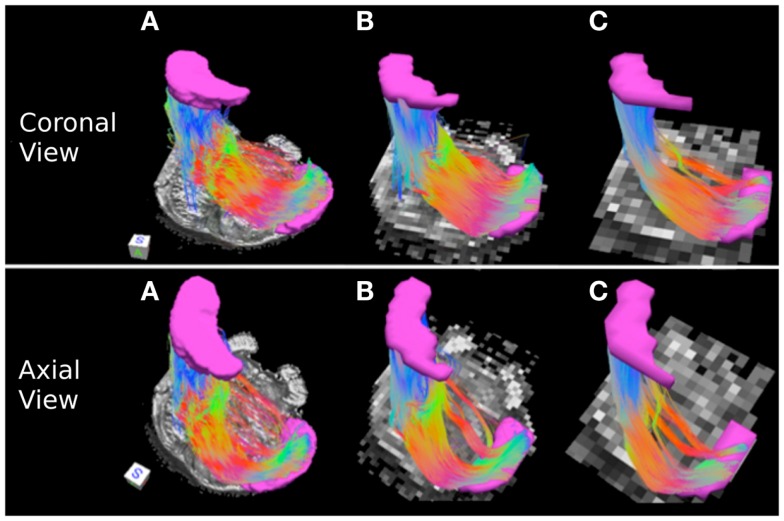
**Middle cerebellar peduncle pathways: (A) 0.333 mm isotropic dataset, (B) 1 mm isotropic dataset, (C) 2 mm isotropic dataset**. An axial slice of the FA maps serves as a background image. Middle cerebellar peduncle (MCP) and the waypoint (cerebral peduncle) mask are depicted in cyan color. Color gradient and three-dimensional cube colors are the same as in Figure 3.

Figure 5 depicts the MCP streamline pathways in the three datasets. Overall the streamline pathways project from the cerebral peduncle to the ipsilateral and contralateral pontine nuclei anteriorly, and to the ipsilateral and contralateral retrolenticular tegmental nuclei posteriorly before entering the MCP. The majority of the streamline pathways in the 0.333 and 1 mm datasets are located predominantly within the anterior extent of the pons consistent with the notion that the cerebellar afferents are more prominent within this region (Figures 5A,B) (Naidich et al., 2008). In the 2 mm resolution dataset, projections to the retrolenticular tegmental nuclei are more prominent that those in 0.333 and 1 mm datasets, as streamline pathways within the posterior extent of the pons are more robust (Figure 5C).

### Medial lemniscus pathways

**Figure 6:**
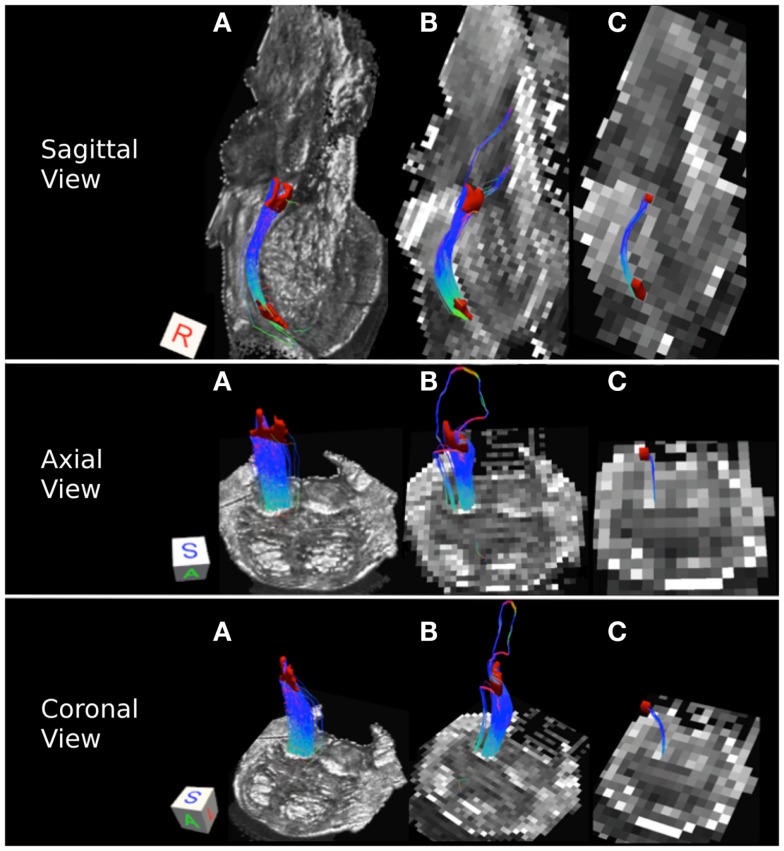
**Medial lemniscus pathways: (A) 0.333 mm isotropic acquisition resolution dataset, (B) 1 mm isotropic resolution dataset, (C) 2 mm isotropic resolution dataset**. An axial slice of the FA maps serves as a background image. Superior and inferior medial lemniscus (ML) regions of interest are depicted in red. Color gradient and three-dimensional cube colors are the same as in Figure 3.

Medial lemniscus pathways carry sensory information from the gracile and cuneate nuclei to the thalamus. Figure 6 represents these pathways in the excised brainstem sample. We note that in the three acquired datasets, the streamline pathways travel within the posterior portion of the pons close to the midline of the brainstem (Figures 6A–C). In the 0.333 mm dataset, the streamlines are tightly packed into a single pathway (Figure 6A), while a secondary lateral streamline pathway is present in the 1 mm data (Figure 6B). This secondary streamline pathway is likely to be comprised of the ALS fibers located in the posterior lateral extent of the brainstem. The streamline pathways in the 2 mm dataset appear to be significantly smaller than those in the 0.333 and 1 mm datasets (Figure 6C). This observation is supported by lower tract volume and edge weight for the 2 mm ML pathways (Table 3). The smaller number of streamline pathways observed in the 2 mm dataset is likely a result of the size of the ROIs used to delineate this pathway. Specifically, as we noted above, the superior and inferior ML ROIs had significantly smaller surface areas as compared with the 0.333 and 1 mm datasets. Insufficient spatial information resulting from volume averaging present in the 2 mm dataset results in loss of gray matter/white matter contrast in the FA image, making it difficult to identify voxels that belong to the ML pathway. Specifically, the gray matter surrounding the ML pathway contributes more as resolution at lower resolution and this results in lower FA values and a smaller number of voxels that can be definitively identified as the ML pathway. Spatial resolution is therefore a limiting factor for the size of the pathways that can be successfully resolved using tractography, especially at lower acquisition resolutions.

### Spatial variability of the inferred streamline pathways as function of acquisition resolution

**Figure 7:**
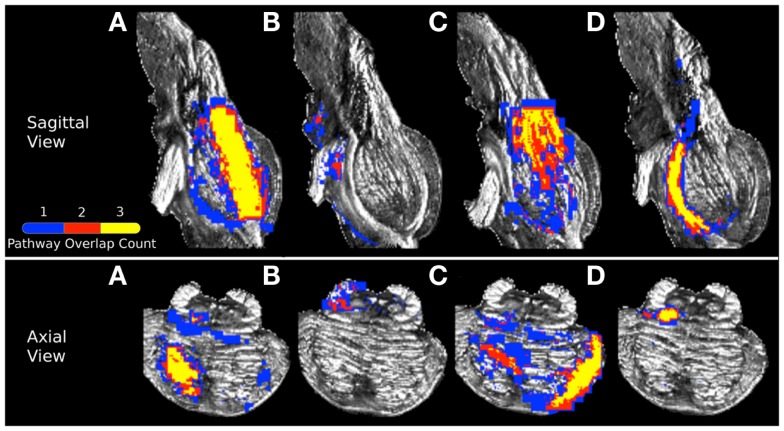
**Visualization of (A) corticopontine/corticospinal pathways, (B) superior cerebellar peduncle pathways, (C) middle cerebellar peduncle pathways, and (D) medial lemniscus pathways inferred from the three acquisition resolution datasets and registered to the 0.333 mm data**. Color gradient represents the amount of spatial overlap between the pathways inferred in from the three acquisition resolutions: yellow colored voxels are common to all three acquisition resolutions, red is common to any two acquisition resolution datasets, and blue is found in only a single dataset.

In order to examine spatial variability of the inferred streamline pathways due to acquisition resolution we registered streamlines inferred in the 1 and 2 mm datasets to the 0.333 mm dataset and examined the percent overlap for each of the four pathways. Table 4 displays the resulting percent overlap values. For the CPT/CST streamline pathways, the percent overlap was 63.12% for the 1 mm dataset and 68.63% for the 2 mm dataset. Figure 7A depicts the overlapping streamline pathways for this pathway. We note that the core of the pathway is common to the three acquisition resolution datasets as indicated by the highest amount of spatial overlap (yellow color in Figure 7 represents voxel values of 3 which implies that the streamline pathways from all three acquisition resolution datasets had these voxels in common). Voxels surrounding the core of the pathway were common to two acquisition resolution datasets (red color in Figure 7 indicates that two datasets had these voxels in common). Most of the voxels comprising the extraneous pathways, such as ML and contralateral projections, were common to a single dataset (as indicated by blue color in Figure 7A). These results show that although the core of the pathway is inferred consistently as resolution is decreased from 0.333 to 1 and 2 mm additional extraneous pathways are also picked up due to volume averaging.

**Table 4:** **Percent overlap between streamline pathways traced in the 0.333 mm dataset and those traced in the 1 and 2 mm datasets**.

Resolution (mm3)	Corticopontine/corticospinal tracts percent overlap (%)	Superior cerebellar peduncle tracts percent overlap (%)	Middle cerebellar peduncle tracts percent overlap (%)	Medial lemniscus tract percent overlap (%)
1 Registered to 0.333	63.12	28.29	70.10	47.31
2 Registered to 0.333	68.73	10.21	67.95	30.44

Figure 7B represents the overlap map for the SCP streamline pathways in the three acquisition datasets. We note that there are no overlapping voxels common to all three datasets (as indicated by the lack of yellow colored voxels). Overall, there was a small amount of overlap between streamlines from the 0.333 mm dataset and 1 and 2 mm data (Table 4). We observed a 28.29% overlap between 0.333 and 1 mm datasets, and 10.21% overlap between 0.333 and 2 mm datasets. This result is consistent with the earlier finding that the SCP streamline pathways have distinct trajectories in the three acquisition datasets (Figure 4).

Figure 7C represents the overlap map for the MCP pathways. We note that similarly to the CPT/CST overlap map, the core of the MCP pathway is common to the three acquisition dataset streamlines, as indicated by the presence of yellow colored voxels (Figure 7C). Specifically, the core of the pathway clusters around the MCP (Figure 7C axial view), and the contralateral cerebral peduncle (Figure 7C sagittal view). In addition, a smaller portion of overlapping voxels can be seen within the contralateral pons (Figure 7C axial view). The small number of overlapping voxels within the contralateral pons is not surprising if we examine the MCP pathways in the three acquisition resolution datasets (Figure 5). This is to be expected because the 0.333 and 1 mm streamline pathways occupy predominantly the anterior portion of the pons, while the 2 mm streamline pathways travel within the posterior portion. These differences in trajectories result in a small amount of overlap within the contralateral pons. Therefore, the majority of overlapping voxels are located within close vicinity of the MCP ROI and within contralateral cerebral peduncle. Specifically, we found a 70.10% overlap between the 0.333 and 1 mm streamlines, and a 67.95% overlap between the 0.333 and 2 mm streamlines (Table 4).

Figure 7D represents the overlap map of the ML streamline pathways. We note that the majority of the voxels common to the streamline pathways in the three acquisition resolutions are clustered within the core of the pathway. However, since the 2 mm dataset inferred streamline pathway is much smaller than those in the 0.333 and 1 mm datasets, the overlapping core (depicted as yellow colored voxels in Figure 7D) accounts for less than a half of all voxels comprising the overlap map. In addition, the percent overlap between the 0.333 and 1 mm dataset streamlines was 47.31%, and only 30.44% between the 0.333 and 2 mm datasets (Table 4). The smaller amount of overlap between the 0.333 and 2 mm streamline pathways results from volume averaging effects present at this lower acquisition resolution.

## Discussion

Tractography studies have provided many valuable insights into white matter organization within the brain (Basser et al., 2000; Johansen-Berg et al., 2005; Catani and Thiebaut de Schotten, 2008; Frey et al., 2008; Rilling et al., 2008; Edlow et al., 2012). Brainstem white matter visualization, however, has proved to be challenging due to the relatively small size of the pathways, high density of their distribution throughout the structure, and image distortions associated with *in vivo* acquisitions (Stieltjes et al., 2001; Habas and Cabanis, 2007; Kamali et al., 2010). The present study aimed to address some of these technical challenges by acquiring high-resolution *ex vivo* data using high field strength MR scanner. In addition, we investigated how the visualization of white mater tracts at the highest currently available acquisition resolution (0.333 mm isotropic) compares with the best *in vivo* acquisition resolution (1 mm isotropic) and the most commonly used *in vivo* acquisition resolution (2 mm isotropic). Our results indicate that although most of white matter streamline pathways examined in this study follow known anatomical trajectories in the three acquisition resolution datasets, volume averaging effects result in inclusion of extraneous pathways in lower resolution datasets. Inclusion of these additional pathways results in enlargement of the inferred fiber bundles reflected by larger tract volumes and edge weight values observed in the present study. Visualizations of white matter pathways in 1 and 2 mm datasets contain less spatial detail and appear more smooth and contiguous as compared with the 0.333 mm pathways (Figures 3–5). Volume averaging effects observed in the present study in 1 and 2 mm datasets were previously reported by *in vivo* tractography studies using similar or lower acquisition resolutions (Stieltjes et al., 2001; Habas and Cabanis, 2007).

Another important result observed in the present study was the finding that the largest changes in overall appearance of the pathways as well as increases in track volumes and edge weight values occur as resolution was decreased from 0.333 to 1 mm (Table 3, with exception of the SCP and the ML pathways). Specifically, we noted a 43% increase in tract volume and a 140% increase in edge weight for the corticopontine/corticospinal pathways as resolution was decreased from 0.333 to 1 mm. Similarly, a 94% increase in track volume and a 2500% increase in edge weight was observed for the MCP pathways. In contrast, when resolution was decreased from 1 to 2 mm, we observed a 3% decrease in track volume and a 56% increase in edge weight for the corticopontine/corticospinal pathways. For the MCP pathways, we observed a less than 2% reduction in track volume and a 2% increase in edge weight values. Large changes in track volumes and edge weight values as resolution is decreased from 0.333 to 1 mm is indicative of substantial loss of spatial information and volume averaging effects. This finding implies that the relative tissue-type contrast loss due to a decrease in resolution from 0.333 to 1 mm results in significantly less accurate modeling of white matter bundles using tractography. As resolution is decreased further from 1 to 2 mm the inferred white matter pathways do not appear to undergo as significant a change as reflected by smaller increases in track volumes and edge weights. This implies that the spatial information present in a 1 mm isotropic dataset does not possess sufficient quality to successfully infer and quantify brainstem white matter pathways.

Two notable exceptions to this trend are the SCP and ML streamline pathways. In particular, for the SCP streamline pathways, a 35% reduction in track volume and a 61% reduction in edge weight was observed as resolution was decreased from 0.333 to 1 mm. These reductions are likely to be due to the reduction in the surface area of the waypoint ROI used to infer these pathways. The waypoint ROI for the 0.333 mm dataset was 581.68 mm^2^, while for the 1 mm dataset its surface area was reduced to 300.24 mm^2^. Surface area reduction for this ROI did not result from poor registration of the ROI from 0.333 to 1 mm dataset, but rather from the fact that a number of voxels along the axial perimeter of the tissue had low diffusion signal due to volume averaging. As a result, following tensor fit, these voxels were assigned low FA values (less than 0.1), which in turn degraded the axial perimeter of the tissue ROI. During the registration process the voxels were classified as background rather than tissue, which resulted in their exclusion from the ROI. This ROI was affected by the volume averaging effects because it contained tissue within the posterior extent of the pons where the MR signal is naturally lower due to presence of many gray matter nuclei.

Similarly, volume averaging effects present at lower acquisition resolutions also affected the size of the ROIs used to delineate ML pathways. In the 2 mm dataset, ML ROIs were less than half the size of the 0.333 mm ROIs (Table 2: 0.333 mm ROIs had surface areas of 78.22 and 113.47 mm^2^, while 2 mm ROIs had surface areas of 27.71 and 55.43 mm^2^). The ML pathway is a fairly small white matter pathway surrounded by gray matter nuclei. As spatial resolution is decreased, surrounding gray matter results in lower FA values due to volume averaging. This effect degrades the tissue-type contrast on the FA image and makes it difficult to identify the ML pathway, reflected by the smaller ML ROIs in our study. Smaller ROIs are a likely contributor to the decreased tract volumes and edge weights observed in our study. We found that the greatest decrease in both tract volume and edge weight was present as acquisition resolution was decreased from 1 to 2 mm (Table 3). Specifically, in the 1 mm dataset, tract volume and edge weight were very close to those observed in the 0.333 mm dataset. However, in the 2 mm dataset we found a 48% decrease in tract volume and a 95.6% decrease in edge weight as compared with the 0.333 mm dataset. These results indicate that the spatial resolution of an acquisition scan may greatly limit the size of white matter pathways that can be successfully inferred using diffusion tractography.

In summary, the results of the present study support the approach that sub-millimeter acquisition resolutions may be necessary to successfully infer and quantify white matter pathways within the brainstem using diffusion-weighted MRI. Previous studies employing 0.6 mm isotropic resolution have found a similar trend where crossing and branching fibers are resolved more efficiently at this high resolution, as compared with 1 and 2 mm acquisitions (Edlow et al., 2012). The present study used a higher acquisition resolution to further test effects of volume averaging in brainstem white matter tractography. Our results indicate that compared with results from highest resolution 0.333 mm isotropic dataset, pathways inferred in 1 and 2 mm datasets include a substantial amount of extraneous pathways that artificially inflate tractography results. Future studies employing even higher spatial and angular acquisition resolutions are necessary to determine the optimal resolution required to accurately visualize brainstem white matter pathways. In addition, histological quantifications of cross-sectional areas and volumes of the pathways should be used as a reference to fully assess volume averaging effects.

## Conflict of Interest Statement

The authors declare that the research was conducted in the absence of any commercial or financial relationships that could be construed as a potential conflict of interest.
